# Topics of Public Interest

**Published:** 1911-03

**Authors:** 


### TOPICS OF PUBLIC INTEREST

---

### Tuberculosis Fighters Will Discuss Results of War

### National Association Meeting at Denver in June—Fall in Death Rate Predicted

SIMULTANEOUSLY with the announcement of its next annual meeting in Denver, June 20 and 21, 1911, the National Association for the Study and Prevention of Tuberculosis presents statistics in a bulletin issued today, which shows the results of the crusade against consumption in the United States for the last ten years.

The seventh annual meeting of the National Association in Denver will be held just before the annual meeting of the American Medical Association in Los Angeles. The tuberculosis workers' convention will be divided into three sections under the direction of Dr. William H. Welch of Baltimore, president. Dr. William Charles White of Pittsburg will be chairman of the Advisory Council of the association, which will meet at the same time. The three sections are, the Sociological, with Alexander M. Wilson of Philadelphia as chairman; the Clinical, with Dr. Charles L. Greene of St. Paul as chairman; and the Pathological, with Dr. William Ophuels of San Francisco as chairman. The report of the executive secretary, Dr. Livingston Farrand, will be incorporated in a statement of the results of the crusade against tuberculosis in the the United States for the last ten years, which will be transmitted to the International Congress on Tuberculosis in Rome next September.

Dr. Farrand's report will show that ten years ago there was only one organization in the United States for the education of the public about tuberculosis, the Pennsylvania Society for the Prevention of Tuberculosis. By September, 1911, the National Association says there will be over 500 such bodies. Ten years ago there were but five special dispensaries or clinics for the examination and instruction of needy tuberculosis patients, three of these being in New York City, one in Boston, and one in Providence. By September, 1911, the United States report will be able to list nearly 400 such institutions. In 1900 there were less than 100 hospitals, wards, and pavilions where tuberculosis patients could be treated, with not more than 6,500 beds all told. The National Association hopes to report by September at least 450 hospitals and sanatoria with an aggregate capacity of at least 30,000 beds.

Commenting on these possibilities, Dr. Farrand says that the educational campaign is directly responsible not only for the great growth in institutional provision but that it will also result in the next ten years in a striking fall in the death rate from tuberculosis. He adds, "What we need most at the moment is more hospitals, more dispensaries, and more visiting nurses. We are working for these definite ends, and the next ten years will show results even more marked than those of the decade just passed."

### The State Board of Regents

On January 25, 1911, the regents of the state of New York canceled thirty qualifying certificates issued to students of the College of the City of New York, it having been shown that the academic credentials issued to the state educational department from that institution were fraudulent, having been sold by a former employe of the college registrar's office. The college authorities took the initiative in searching out the fraud.

### The Physicians' Mutual Aid Association

The forty-second annual meeting of the New York Physicians' Mutual Aid Association was held January 17, 1911. The report of the treasurer showed the finances to be in a satisfactory condition. The present membership is 2,074, a gain of 117 for the fiscal year. Physicians in all parts of New York state are eligible and the membership should be increased greatly, to correspond with the benefits received.

Camphor in Large Doses in Pneumonia.—Leonard Weber, New York (*Medical Record*, January 21), advocates the use in pneumonia of injections of camphor in oil, made aseptically, with the needle thrust well down into the cellular tissue. Thus given there will be no pain, local swelling, or irritation of bladder, kidneys, or stomach. The author details a case of pneumonia in which there were evidences of intense pneumococcic septicemia, in which camphor was used successfuly after this method, and recovery occurred.

